# Cumene Contamination in Groundwater: Observed Concentrations, Evaluation of Remediation by Sulfate Enhanced Bioremediation (SEB), and Public Health Issues

**DOI:** 10.3390/ijerph17228380

**Published:** 2020-11-12

**Authors:** John P. Herman, Lauren Redfern, Christopher Teaf, Douglas Covert, Peter R. Michael, Thomas M. Missimer

**Affiliations:** 1 Emergent Technologies Institute, U. A. Whitaker College of Engineering, Florida Gulf Coast University, Fort Myers, FL 33901, USA; jpherman@eagle.fgcu.edu (J.P.H.); pmichael@fgcu.edu (P.R.M.); 2 U. A. Whitaker College of Engineering, Florida Gulf Coast College, Fort Myers, FL 33965, USA; lredfern@fgcu.edu; 3 Institute for Science & Public Affairs, Florida State University, Tallahassee, FL 32306, USA; cteaf@fsu.edu; 4 Hazardous Substance and Waste Management Research, Tallahassee, FL 32309, USA; dcovert@hswmr.com

**Keywords:** isopropylbenzene (cumene), gasoline, groundwater contamination, remediation, public health risk

## Abstract

Isopropylbenzene (cumene) is commonly encountered in groundwater at petroleum release sites due to its natural occurrence in crude oil and historical use as a fuel additive. The cumene concentrations detected at these sites often exceed regulatory guidelines or standards for states with stringent groundwater regulations. Recent laboratory analytical data collected at historical petroleum underground storage tank (UST) release sites have revealed that cumene persists at concentrations exceeding the default cleanup criterion, while other common petroleum constituents are below detection limits or low enough to allow natural attenuation as a remediation strategy. This effectively makes cumene the driver for active remediation at some sites. An insignificant amount of research has been conducted for the in-situ remediation of cumene. Sulfate Enhanced Biodegradation (SEB) is evaluated in a field case study. The results from the field case study show an approximate 92% decrease in plume area following three rounds of SEB injections. An additional objective of this research was to determine the cumene concentration in fuels currently being used to determine future impacts. A review of safety data sheets from several fuel suppliers revealed that cumene concentrations in gasoline are reported typically as wide ranges due to the proprietary formulations. Several fuels from different suppliers were analyzed to determine a baseline of cumene concentration in modern fuels. The results of the analysis indicated that cumene accounts for approximately 0.01% (diesel) to 0.13% (premium gasoline) of the overall fuel composition. Cumene generally is considered to be of low human health toxicity, with the principal concern being eye, skin, and respiratory irritation following inhalation of vapors in an occupational setting, but it has been regulated in Florida at very low concentrations based on organoleptic considerations.

## 1. Introduction

Cumene (isopropylbenzene) is a volatile organic compound (VOC) consisting of an aromatic hydrocarbon with an aliphatic substitution [1]. The compound is not known to be a human carcinogen due to insufficient evidence of carcinogenicity in human studies. However, it is judged as “reasonably anticipated” to be a human carcinogen based on findings of studies with experimental animals. There is evidence that the metabolism of cumene in humans is similar to that of the experimental animals [2].

Cumene is produced in high volumes in the United States with the majority of the chemical used as an intermediate in the synthesis of phenol and acetone [2]. It is also a natural constituent in crude oil and is used as a fuel additive for diesel, various grades of gasoline, and heating oil [3]. Due to its presence in petroleum products, it is commonly identified in petroleum-impacted groundwater from Underground Storage Tank (UST) discharges. Data from several subsurface investigations in South Florida indicate that cumene is rather recalcitrant and not easily attenuated by natural processes, while other sources report that cumene persistence and degradation vary with site-specific conditions [4,5,6]. Site assessments have revealed cumene persisting in the subsurface at concentrations exceeding regulatory guidelines in sites over 30 years following release discovery.

Cumene is commonly identified at petroleum-impacted sites when the released product type is unknown or is suspected to be used oil. In these situations, analysis according to Florida Administrative Code (FAC) Chapter 62-780, Table D is required. Table D includes analysis of priority pollutant volatile and extractable organics [7]. At many sites, cumene was detected during such analyses at concentrations exceeding the current Florida Administrative Code (FAC) Chapter 62-777 Natural Attenuation Default Concentration (NADC). Active remediation or pursuit of Risk Based Corrective Action (RBCA) is required when a contaminant is detected above the NADC [8]. Other common petroleum constituents often are detected at these sites as well, but may occur at concentrations low enough to allow for natural attenuation monitoring as a remediation strategy. However, the NADC for cumene is set at 8 μg/L [7,8], predicated on extension of the organoleptic-based Groundwater Cleanup Target Level (GCTL). This is a very restrictive target in comparison to many other petroleum hydrocarbons, making it a common driver for remediation or other mitigation approaches. There has not been a significant amount of research conducted on the in-situ remediation of cumene. The lack of research may be in part due to the fact that the standard for cumene set by most state regulatory agencies is either set considerably higher than that in Florida or, in most cases, the substance is unregulated. For early assessment of petroleum contamination sites in many jurisdictions, cumene concentration was not measured.

Several petroleum release sites in Florida with documented cumene exceedances of state guidelines were identified through a search of the Florida Department of Environmental Regulation (FDEP) Oculus Database. Remediation data were available for only a few of the identified sites. Remedial techniques including In Situ Chemical Oxidation (ISCO), Sulfate Enhanced Biodegradation (SEB), Air Sparging/Soil Vapor Extraction (AS/SVE), groundwater extraction, or Monitored Natural Attenuation (MNA) were employed with varying degrees of success. Of the remedies reviewed, SEB was found to be among the most effective. This paper considers whether cumene can be remediated using sulfate-enhanced bioremediation and, in addition, assesses the foundation of the Florida criterion for remediation of cumene.

## 2. Background

### 2.1. Cumene as a Groundwater Contaminant

#### 2.1.1. Environmental Concentrations of Cumene in Groundwater

High levels of cumene have been found in groundwater near industrial sites and in industrial effluents. Concentrations of 360 [9] and 1581 μg/L [10] have been reported in groundwater near underground storage tanks, as high as 700 μg/L near outboard motor operations [11], and up to 54 μg/L near coal gasification facilities [12]. Cumene has been detected at lower levels (usually less than 5 μg/L) in groundwater not adjacent to a known industrial or a contaminated site [4,13].

#### 2.1.2. Cumene Remediation in Groundwater

Several techniques have been investigated for the removal and remediation of cumene. Due to its tendency to sorb to soils (K_oc_ value of 3.45), chemical oxidation is not recommended for cumene treatment in groundwater [14]. Cumene can be removed by sorption to activated carbon or resin (USEPA). Additionally, cumene is well-established to be biodegradable; phytoremediation [15] and bioremediation [16] have been proven to be effective and relatively cost-efficient remediation approaches. In addition, cumene biodegradation can occur in both anaerobic and aerobic conditions [17,18,19,20,21]. Cumene biodegradation occurs by dioxygenase, and a cumene dioxygenase has been isolated and characterized from a *Pseudomonas fluorescens* strain using cumene as its sole carbon source [22].

### 2.2. Cumene in Fuels

Cumene concentrations in petroleum fuels are not generally reported or are simply given as ranges due to proprietary formulations. Table 1 below shows ranges of cumene concentrations provided by various gasoline suppliers that report the chemical composition in their Safety Data Sheet (SDS) or Material Safety Data Sheet (MSDS). The table is sorted by cumene concentration (low to high). The average cumene concentration in gasoline (various grades) and premium diesel was reported to be 0.3% by volume and 0.86% by weight, respectively. Crude oils typically contain approximately 0.1% cumene by weight but can contain as much as 1.0% [3]. The data in Table 1 indicate that cumene can account for up to a 10% share of the gasoline product formulation.

Due to the variability of reported concentrations, baseline cumene concentrations of modern fuels were measured to assess potential impacts from fuel discharges to groundwater in terms of cumene remediation. This baseline analysis is outlined in subsequent sections of this article. Selected properties of cumene are provided in Table 2.

### 2.3. Current Cumene Regulations

The Florida regulatory guideline for cumene is set significantly lower than most other states. Groundwater standards for cumene in Table 3 were identified in regulatory rules for 14 state agencies. The compound is assumed to be unregulated in all other states. Standards for countries other than the United States were not evaluated. Cumene standard concentrations are presented, ranked from low to high.

### 2.4. Sulfate Enhanced Biodegradation (SEB)

**Figure 1:**
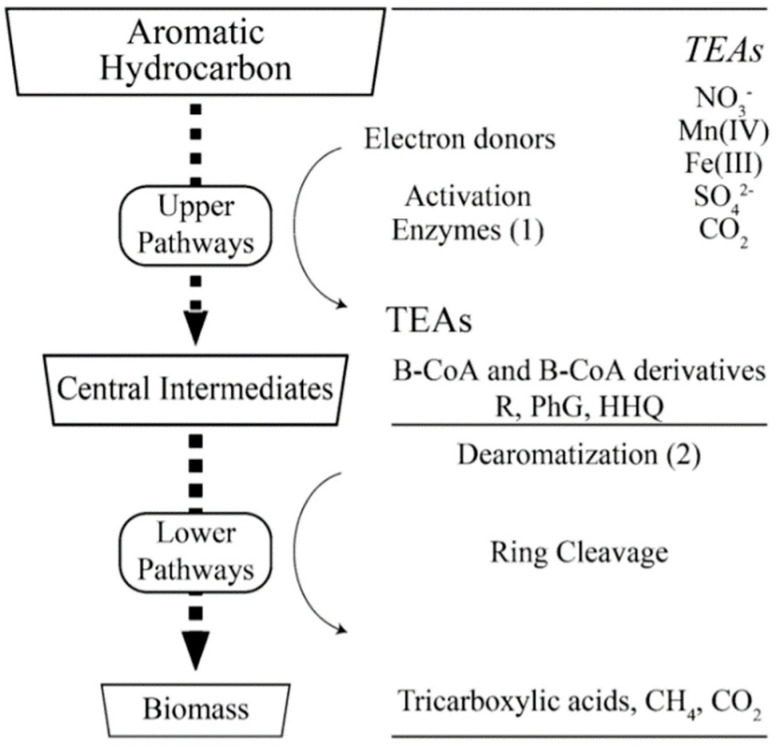
Bacterial pathways for aromatic hydrocarbon anaerobic degradation [47].

Sulfate enhanced bioremediation (SEB) is a proven remediation strategy for petroleum impacted groundwater. This remedial process works by applying an amendment of sulfate to an impacted medium that is currently using or can potentially use sulfate as a terminal electron acceptor (TEA). Native sulfate reducing bacteria (SRB) reduce the sulfate compound while simultaneously oxidizing the petroleum hydrocarbon. A general schematic showing the pathways of hydrocarbon degradation by bacteria under anaerobic conditions is presented in Figure 1. The upper pathway includes the route from the original compound to central intermediate substances, which still contain the aromatic ring but with the benzene nucleus chemically destabilized. The lower pathway begins with dearomatization and ring cleavage, which produce products that can be metabolized by bacteria [47].

Site characterization is required to determine the identity and prevalence of the current electron acceptor. However, anaerobic bioremediation by sulfate reduction is the dominant terminal electron accepting process [48], and it accounts for approximately 70% of the natural biodegradation capacity [49]. Generally, in-plume (especially downgradient) concentrations of sulfate will be close to or below laboratory detection limits, while background concentrations will be considerably higher if sulfate is the electron acceptor.

Sulfate is highly water-soluble and does not readily sorb to soil particles, increasing the quantity of the TEA available to sulfate reducing bacteria [14]. It is also advantageous that SRBs are considered prolific and are capable of metabolizing a broad spectrum of petroleum hydrocarbons [48]. More than 220 species of 60 genera of SRB have been identified [50]. However, one potential limitation to this technology is often the secondary drinking water standard set for sulfate of 250 mg/L, which is based on taste, rather than potential health impacts [51].

## 3. Materials and Methods

### 3.1. Baseline Cumene in Modern Fuels

Gasoline and diesel samples were collected at gasoline service stations to establish a baseline of cumene concentrations in modern fuels. To maintain brand anonymity, the samples were labeled as one through five, with each number representing a different fuel supplier. A sample of regular (87 octane), mid-grade (89 octane), and premium (93 octane) gasoline was collected from each of the five fuel suppliers. In addition, a diesel sample was collected from Gas Stations #2, #4, and #5, and an ethanol-free sample was collected from Gas Station #3. The samples were placed into ANSI/ASTM/CARB-compliant and EPA-approved polyethylene gasoline canisters with sufficient volume (minimum 0.5 gallon) to minimize cross-contamination from other various fuel grades. Within 15 min of sample collection, a 2 mL aliquot from each gas can was transferred into a 9 mm clear glass autosampler vial using pipets. The sample vials were then immediately placed on ice and analyzed within 24 h of collection.

The samples were analyzed utilizing a Shimadzu GCMS-QP2020 NX EI Gas Chromatograph Mass Spectrometer (GC-MS) equipped with a direct injection tower and a 30 m long by 0.25 mm inner diameter capillary column. The analysis was performed in general accordance with EPA Method 8260. It should be noted that a purge-and-trap autosampler and concentrator is typically used to analyze VOCs. However, EPA Method 8260 allows for the sample introduction to be performed by direct injection when volatile concentrations are in excess of 10,000 μg/L [52]. This method is applicable because cumene concentrations in the undiluted samples were expected to be approximately 0.3% (3,000,000 μg/L) based on an initial literature review. The GC-MS was calibrated with cumene reference standards prior to analyzing any of the fuel samples to minimize the potential for mischaracterization of cumene with similar analytes.

### 3.2. Field Case Study

Sulfate Enhanced Bioremediation (SEB) was chosen as the remediation technology at a petroleum impacted site in Broward County, Florida (USA) after site characterization was performed. The site assessment and cleanup for the case study is state-funded under Florida’s Petroleum Restoration Program (PRP) and is being performed by an FDEP-contracted environmental consultant. In addition to the delineation of the petroleum contamination, site characterization also included analysis of various bioremediation and geochemical parameters such as common electron acceptors and metabolic byproducts in the plume and background monitoring wells. Specifically, the analysis included iron (total and dissolved), manganese (total and dissolved), alkalinity, biological oxygen demand (BOD), total organic carbon (TOC), nitrate, nitrite, total nitrogen, total Kjeldahl nitrogen (TKN), orthophosphorus, sulfate, sulfite, sulfide, heterotrophic plate count, hydrogen sulfide, carbon dioxide, and methane. Sulfite was analyzed in the field using a Hach SU-5 titration test kit. The remaining analysis was performed by a commercial laboratory using appropriate laboratory or EPA Methods. Sulfate was determined to be the active TEA based on the results. Following site characterization, the list of bioremediation parameters analyzed for subsequent remedial action monitoring events narrowed to include only alkalinity, sulfate, sulfite, and sulfide. VOCs, including benzene, toluene, ethylbenzene, xylenes, methyl tert-butyl ether (MTBE), and cumene were measured during each sampling event. The groundwater samples for VOC analysis were prepared using EPA Method 5030B and analyzed according to EPA Method 8260B. A commercial laboratory made the measurements on a GC-MS using a purge and trap unit before the instrument.

The baseline in-plume cumene concentrations ranged from 2.0 to 20 μg/L (average 9.74 μg/L) with NADC exceedances in four monitoring wells. The estimated plume area using baseline concentrations was 399.6 m^2^ with a vertical extent ranging from the top of the surficial aquifer at 1.5 m below land surface (bls), down to 10.7 m bls. Prior to the baseline analysis, cumene was the only contaminant identified at concentrations exceeding NADC standards. However, the baseline assessment also revealed benzene exceeding its respective NADC of 100 μg/L in one of the monitoring wells (170 μg/L). This field study was used as an example in Best Available Practice to remediate cumene contamination of groundwater.

Sulfate was introduced into the impacted groundwater as an aqueous blend of magnesium sulfate (Epsom salt) with water in an initial pilot study. The initial goal was to raise the in-plume sulfate concentrations from non-detect to approximately 200 mg/L. To accomplish this, approximately 568 L of an estimated 589 mg/L sulfate solution was injected at 12 separate points from 1.5 to 10.7 m bls (6816 L total injection volume). The solution was injected via a direct push drill rig using hollow steel rods and a pressure-activated injection probe. The injection was performed under low pressure (generally less than 2.1 bar) to ensure uniform distribution and minimal channeling. The depth to water and several geochemical parameters were monitored at various adjacent wells during the injection event to measure influence of the injection and any groundwater mounding effects.

**Figure 2:**
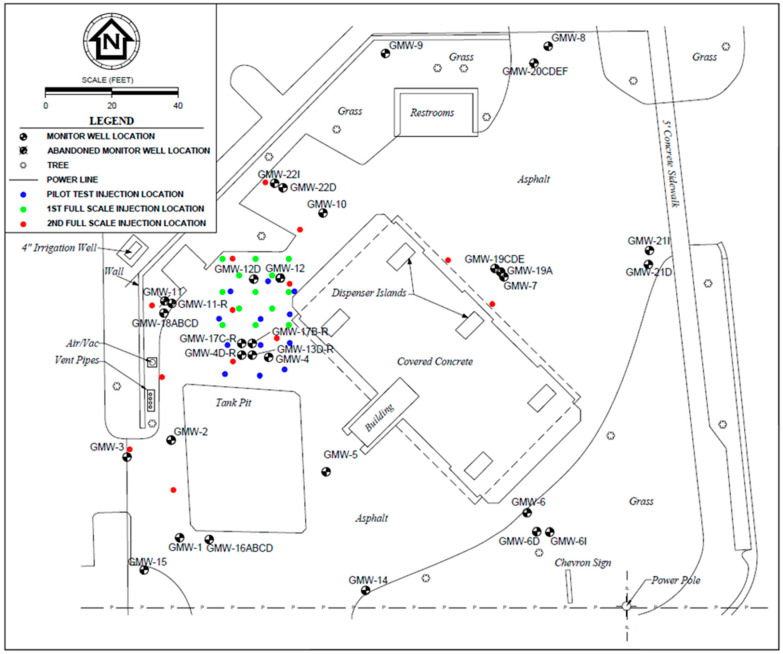
Case study general site plan with injection point locations.

A total of three (3) injection events with sulfate were performed at the described remediation site. A site plan showing the current layout of the facility with monitoring well and injection point locations as well as other pertinent features is illustrated in Figure 2. The injection points for the pilot test and the second event (1st full-scale injection) were spaced approximately 1.8–3 m (6–10 ft) apart within the estimated cumene NADC plume area. The injection point spacing for the third event (2nd full-scale injection) was increased to address monitoring wells with historical exceedances (to reduce the potential for future rebound). The 1st full-scale and 2nd full-scale injection events were performed following the same general procedure as the pilot test event with an additional injection point (13 total) and modifications to the injectant volume (757 L for each injection point) and sulfate concentration (approximately 3000 mg/L for the 1st full-scale injection, and 6000 mg/L for the 2nd full-scale injection). The injection events were performed approximately 16 months apart to allow time to evaluate remediation efficacy. The monitoring schedule varied from monthly during the pilot testing activities to quarterly or semi-annual thereafter. A gap in monitoring occurred between the pilot testing and 1st full-scale injection due to the time necessary to prepare and obtain approval for the remedial action plan and new purchase order.

### 3.3. Health Risk Assessment of Cumene

An assessment of the health issues related to cumene exposure was conducted via a literature survey (see Section 4.3). Specific health studies were summarized to draw conclusions concerning exposure risk in the context of Florida regulations.

## 4. Results

### 4.1. Baseline Cumene in Modern Fuels

The cumene concentrations in fuels analyzed ranged from 125,802 (4-Diesel) to 1,323,250 μg/L (1-Premium). The margin of error is estimated to be +/− 3.5% with a confidence level of 95% based on the results of five separate runs on the same sample. Data from the GC/MS analysis of the fuels are shown in Table 4. The individual chromatograms are presented in the Supplemental Materials as Figures S1–S21. A second cumene standard was used to analyze samples collected from Gas Stations #4 and #5 (analyzed at a later date than Gas Stations #1, #2, and #3).

The results from the samples collected from Gas Station #1 show an increasing cumene concentration with increasing octane rating. For Gas Station #2, the cumene concentrations in the regular and midgrade are fairly similar, while the concentration in the premium fuel was significantly higher (approximately 41% higher). The diesel samples (2-Diesel, 4-Diesel, and 5-Diesel) exhibited the lowest concentrations of cumene. The chromatogram for the diesel samples (Supplemental Information) are unique in comparison to the gasoline chromatograms. The analysis of the fuels from Gas Station #3, #4, and #5 showed little variability of cumene concentrations between the different grades. However, the cumene concentrations in the samples collected from Gas Station #3 were much higher than the concentrations from the samples collected from Gas Stations #4 and #5.

### 4.2. Case Study

**Figure 3:**
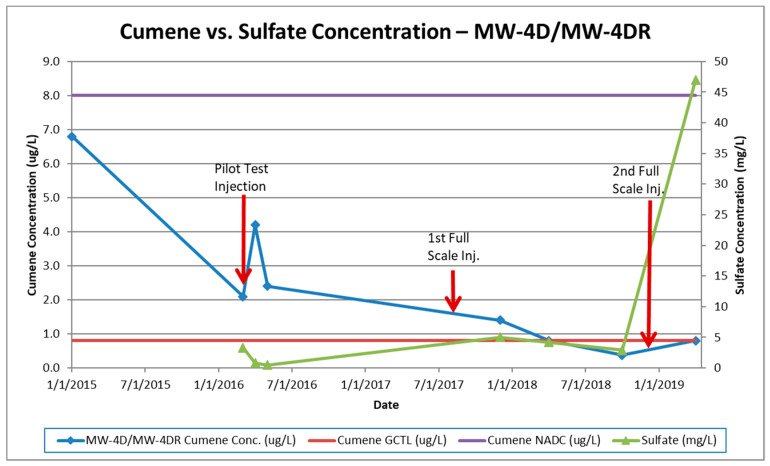
Cumene vs. sulfate concentrations in MW-4D/MW-4DR.

**Figure 4:**
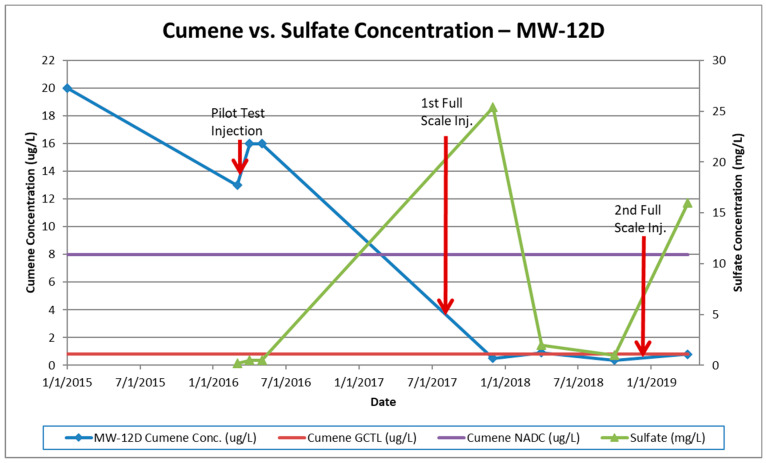
Cumene vs. sulfate concentrations in MW-12D.

**Figure 5:**
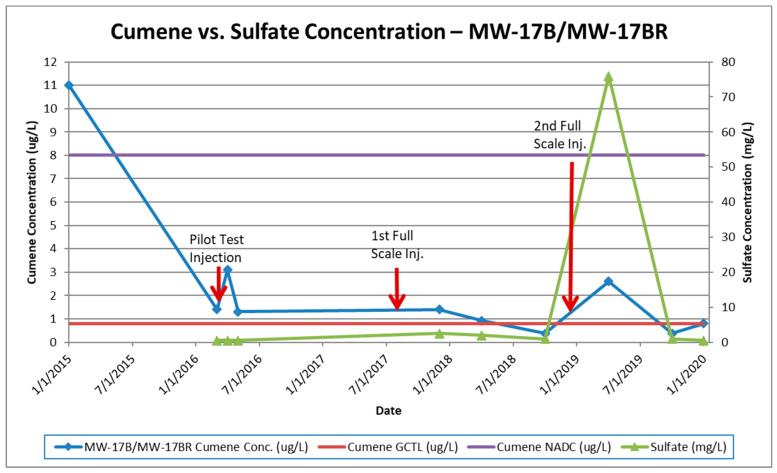
Cumene vs. sulfate concentrations in MW-17B/MW-17BR.

The first round of injections was conducted during the pilot test activities as described. The initial results showed a spike in contaminant concentrations, which is attributed to the mobilization of adsorbed contamination from the saturated soil source zone into the groundwater, followed by a steady decrease. Only minimal increases in sulfate concentrations in the plume were observed. The monitoring event performed after the 1st full-scale injection showed minimal to moderate increases in sulfate concentrations with slight cumene decreases. Major increases in in-plume sulfate concentrations (up to 370 mg/L) were observed after the 2nd full-scale injection. Cumene concentrations decreased in most monitoring wells immediately following the third injection with some wells showing slight increases. Large decreases in both sulfate and cumene concentrations were observed approximately 180 days following the third injection event. These concentrations reduced further in the subsequent monitoring event (sulfate concentrations at or below background concentrations). The regulatory GCTL criterion for cumene was then only exceeded in one monitoring well and the estimated plume size is approximately one-thirteenth of its original size (now roughly 31.2 m^2^). Figure 3, Figure 4 and Figure 5 illustrate cumene and sulfate concentrations over time for the key monitoring wells at the site during the injection cycles. These wells were selected for graphical representation because of the high initial and persistent cumene concentrations observed during remediation.

### 4.3. A Review of Health Impacts of Cumene

Cumene presents a generally low toxicity hazard profile [53]. Reports concerning adverse effects on humans following exposure to cumene primarily are associated with long-term exposure to cumene vapors under occupational conditions. Less than half of a worker population exposed for 7–10 years to unspecified levels of cumene vapors experienced alterations in hepatic enzymatic activity, increased bilirubin concentrations, changed lipid metabolism, altered liver and hepatobiliary functions, and dyskinesia (involuntary muscle movements [54]). Cumene at high concentrations in air can be a skin and eye irritant, and exposure to vapors at high concentrations may cause dizziness, slight incoordination, and unconsciousness, depending on the exposure frequency and duration [53,54]. Protective concentrations for occupational circumstances have been set by the National Institute for Occupational Safety and Health (NIOSH) and the Occupational Safety and Health Administration (OSHA) at 245 mg/m^3^ (50 parts per million, ppm [55]). Consistent with other alkylbenzenes (e.g., ethylbenzene, toluene, xylene), and countless other substances, cumene may be irritating to skin, eyes, the respiratory system, and the central nervous system upon sufficient exposure.

In Florida, while there is no formal Drinking Water Standard for cumene, the assessment and management of cumene in groundwater are regulated under Chapter 62-780, Florida Administrative Code (F.A.C.) [8] and Chapter 62-777, F.A.C. [7] via an “organoleptic” Cleanup Target Level (CTL) of 0.8 µg/L. Organoleptic CTLs, as with other “secondary” water quality standards, are based on avoidance of taste, odor, or staining issues. That value is far below any value that would be based on its potential to cause adverse toxicological effects. For example, a protective health-based criterion of 700 µg/L for human consumption is presented by FDEP in Table F of Chapter 62-780, and that same concentration is published as a safe level by the Florida Department of Health [56]. USEPA also developed an analogous health-based criterion for cumene in residential tap water in the Regional Screening Level (RSL) process. That USEPA [57] value is 450 µg/L for protection of human health under domestic residential uses, including drinking water. Furthermore, the USEPA Office of Water provides a Drinking Water Equivalent Level (DWEL) of 4000 µg/L, for lifetime drinking water exposure to cumene [58].

Health-based groundwater screening levels such as those discussed here typically are based on toxicological guidance values—e.g., oral Reference Doses (RfDs) and inhalation Reference Concentrations (RfCs) that often are derived from animal studies. That is indeed the case for cumene. The oral RfD of 0.1 mg/kg·day is based on observed increased kidney weight in female rats, and the inhalation RfC of 4 × 10^−1^ mg/m^3^ (0.4 mg/m^3^; 400 µg/m^3^) is based on increased kidney weights in female rats and increased adrenal weights in male and female rats [59]. Conservative uncertainty factors were applied to the animal studies for extrapolation to human health decisions (i.e., RfD, RfC). Human exposure studies are not available from which to develop reliable toxicological guidance values.

The International Agency for Research on Cancer (IARC) classified cumene in Group 2B, possibly carcinogenic to humans [60], and the U.S. National Toxicology Program [2] concluded that cumene was “reasonably anticipated to be a human carcinogen”. However, potential carcinogenic effects do not form the basis for any of the protective human health-based water, soil, or air criteria cited herein. Furthermore, NTP drew no conclusion regarding exposure levels at which cumene may or may not exert the described carcinogenic effects, established no potency estimates, and concluded that it is not mutagenic or genotoxic in most standard in vitro and in vivo assays [2]. The fact that cumene is not genotoxic/mutagenic strongly suggests that the substance acts in a threshold manner, and that the numerical health-based criteria developed by various agencies (e.g., RfD, RfC, health-based standards) are indeed protective of human health.

With respect to potential off-gassing from groundwater to overlying soil and then into ambient air or into indoor air space of buildings over groundwater, according to USEPA’s Vapor Intrusion Screening Level (VISL) Calculator, cumene has a target groundwater concentration of 850 µg/L for protection against possible vapor intrusion exposure concentrations. That value is derived by back-calculating from safe indoor air screening levels established as protective health-based concentrations below which no further action or investigation is warranted [61].

## 5. Discussion

### 5.1. Baseline Cumene in Modern Fuels

Analytical results from the samples collected showed cumene concentrations varying from 125,802 (4-Diesel) to 1,323,250 μg/L (1-Premium). The trend of increasing cumene concentration with increasing octane rating for Gas Station #1 fuels indicates that cumene is likely used as an octane boosting additive for the mid and premium grade gasoline blends at this station. The difference in concentrations between the mid-grade and premium gasoline for Gas Station #2 indicates that the compound is an octane booster for the premium gasoline only. The small variability of cumene concentrations in the fuels from Gas Station #3 suggests that the compound is either added to each grade at similar concentrations or it is naturally occurring in the source crude oil at those concentrations. The results from Gas Stations #4 and #5 also show very little variability in cumene concentrations between the different octane grades. However, the average cumene concentrations in Gas Stations #4 and #5 (418,788 and 456,855 µg/L, respectively) are much lower than that of Gas Station #3 (1,046,704 µg/L). Additionally, cumene concentrations in 2–Regular and 2–Mid Grade are similar to cumene concentrations of all gasoline samples collected from Gas Stations #4 and #5. This may indicate that cumene concentrations in the range of 400,000–500,000 µg/L are present from natural sources. Concentrations reported in this study are considerably lower than values found in the literature (0.3% for gasoline and 0.86% for diesel). However, those values represent research conducted in the 1980s. Gasoline formulations change over time due to technological advancements and changing environmental regulations, which may explain the variability observed. Despite the lower concentrations in modern fuels, they are not insignificant given their persistence in the subsurface, coupled with restrictive regulatory criteria.

### 5.2. Effectiveness of Enhanced Bioremediation with Sulfate

**Figure 6:**
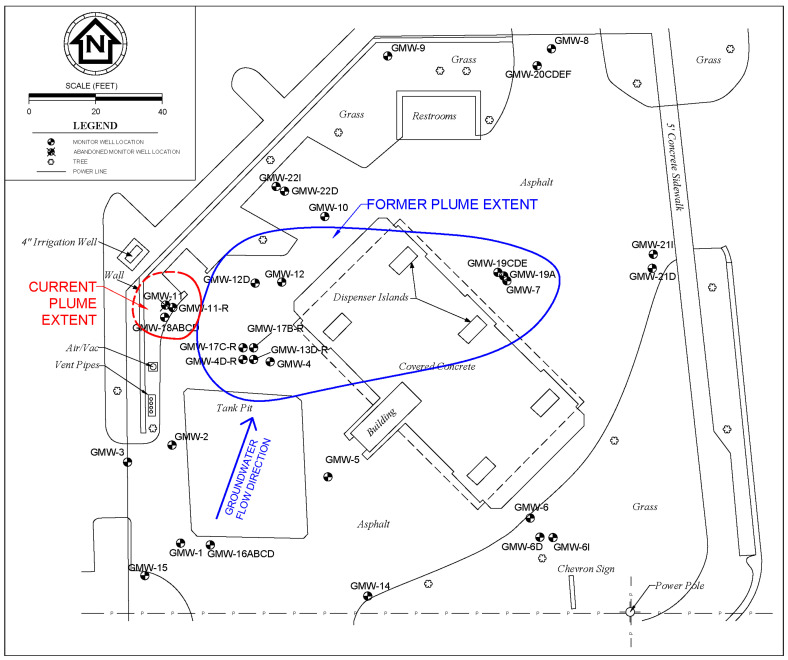
Site plan with estimated groundwater flow direction and plume extents.

The SEB successfully reduced cumene concentrations in this case study. The plume size was decreased by approximately 92%, and the site is on track to qualify for a clean closure (complete site rehabilitation). Clear increases in in-plume sulfate concentration were observed following the second and third injection events with the sulfate solution. In some cases, sulfate concentrations exceeded the sulfate secondary drinking water standard (250 mg/L). However, within two quarters following each injection event, sulfate concentrations had returned to background or undetectable quantities. This quick decline indicates that sulfate was likely the acting TEA. Based on the rate of TEA utilization, the time between injection events could have been decreased to approximately 6 months to condense the remediation time. All monitoring wells within the original plume extent tested below CTLs approximately 3.5 years after the pilot test injection. Currently, cumene is only detected in one shallow monitoring well, which is upgradient from the source area. That monitoring well was recently abandoned and replaced due to persistent observations of debris (roots) and intermittent turbidity issues noted during sampling. The replacement monitoring well has been sampled only once since installation, resulting in a slight cumene concentration. This level was likely caused by a disruption of the soils during the well installation activities. It is postulated that residual cumene mass adsorbed to saturated zone soils was temporarily released into the groundwater, similar to what was observed following the pilot test injection event. The estimated groundwater flow direction and a comparison of current and former plume extents (based on the GCTL for cumene) is illustrated on Figure 6. It should be noted that the current plume lacks complete definition (plume line is dashed where inferred to the west). The offsite irrigation well to the northwest was sampled for cumene with no detections. However, the irrigation well was screened at a deeper interval. A shallow monitoring well is proposed here to provide adequate delineation if subsequent replacement well sampling results in persistent cumene exceedance.

Due to its success at this site, pilot-scale SEB is being implemented at a few other sites in South Florida to evaluate its effectiveness for remediation of other VOCs and Polycyclic Aromatic Hydrocarbons (PAHs). Initial results indicate that SEB is successfully remediating other VOCs such as benzene, toluene, ethylbenzene, xylenes, and some cumene isomers, but it may not be appropriate for general PAH-driven remedial activities.

It is likely that the added sulfate stimulates the growth of sulfate-reducing bacteria, which are the primary cause of the cumene breakdown. More research needs to be conducted on the bacterial genomics of specific groundwater species that cause the cumene breakdown.

### 5.3. Public Health Issues and Mandated Cumene Remediation in Florida

Because of the limited human health concern regarding cumene, and because the default groundwater cleanup guideline is not a health-based value, potentially responsible parties and FDEP remedial project managers should carefully evaluate risk-based options when cumene in groundwater is the driving substance of a remediation project. Perhaps the Florida Department of Environmental Protection should re-evaluate the very low concentration used to trigger remedial activities, especially when the concentrations of other contaminates do not exceed remediation guidance. Since cumene is degraded by naturally occurring, anaerobic bacteria, remediation on sites with low concentration, even above the regulatory limit, should be evaluated using risk-based criteria. At locations where human exposure is unlikely, natural attenuation should be given consideration as a remedial strategy.

## 6. Conclusions

Cumene (Isopropylbenzene) is a naturally occurring substance in unrefined petroleum and is used as an additive in medium and high-grade gasoline to enhance octane. It is regulated in water in 14 of 50 states with action standards ranging from 5 to 840 µg/L. Because of the paucity of data with regard to impacts on human health, the clean-up guidelines used by the states that have them are not based on public health considerations. Cumene is considered to have low toxicity to humans but is regulated at very low concentrations in Florida (8 µg/L).

An effective means of remediating cumene in groundwater is Sulfate Enhanced Biodegradation (SEB). Addition of sulfate to groundwater promotes growth of various anaerobic bacteria that cause breakdown of cumene into harmless constituents. Typical remediation methods for other gasoline components are not effective for use on cumene.

Based on a generally low potential impact to human health, a different, risk-based approach to mandating remediation should be considered, especially at sites where potential contact with humans is minimal. Most groundwater in Florida is anoxic and contains the specific types of anaerobic bacteria used in the SEB remediation strategy. Therefore, natural attenuation is a remediation strategy that should be considered in low risk sites in lieu of performing active remediation.

## Figures and Tables

**Table 1:** Cumene concentration ranges reported by various fuel suppliers Material Safety Data Sheet/Safety Data Sheet (MSDS/SDS) [23,24,25,26,27,28,29,30].

Gasoline Brand Name/Grade	% Cumene Conc.	Reference
Shell–All Grades Unleaded	0–0.5%	US Oil MSDS 2012
ExxonMobil–Unleaded with Ethanol	0–1%	Canada Imperial MSDS 2009
Gulf–All Grades Unleaded	0–1%	Gulf SDS 2018
Sunoco–87 Unleaded	0–1%	Sunoco SDS 2015
Citgo–All Grades Unleaded	0–4%	Citgo SDS 2018
Marathon–All Grades Unleaded	0–4%	Marathon SDS 2018
Petrocom–All Grades Unleaded	0–5%	Petrocom MSDS 2008
Valero–All Grades Unleaded	0–5%	Valero SDS 2014
Flint hills–All Grades Unleaded	0–10%	Flint Hills MSDS 2012

**Table 2:** Select properties of cumene.

Property	Value	Reference
Cas number	98-82-8	[31]
Molecular formula	C H	[31]
Molecular weight	120.191 g/mol	[31]
Color	Clear/Colorless	[32]
Odor	Sharp, penetrating, aromatic	[32]
Boiling point	152.4 °C	[31]
Flash point	36 °C	[31]
Melting point	−96.01 °C	[31]
Vapor pressure (at 25 °C)	0.61 kPa	[31]
Water solubility (at 25 °C)	0.050 g/kg	[31]
Octanol/water partition coefficient, log k	3.66	[31]
Density (at 20 °C)	0.8615 g/cm^3^	[31]
Henry’s law constant, k (at 20 °C)	1.466 kPa-m^3^/mol	[31]

**Table 3:** Regulatory groundwater standard for cumene by state.

State	Cumene Criterion (µg/L)	Reference
New York	5	[33]
Florida	8	[34]
Maryland	45	[35]
Deleware	66	[36]
North Carolina	70	[37]
Minnesota	300	[38]
Maine	450	[39]
Kansas	451	[40]
Illinois	700	[41]
Iowa	700	[42]
New jersey	700	[43]
Michigan	800	[44]
New Hampshire	800	[45]
Pennsylvania	840	[46]

**Table 4:** Cumene concentration in various modern fuels.

Substance	Peak #	R. Time	I. Time	F. Time	Area	Cumene (µg/L)	Cumene (%)
Cumene Standard #1	1	7.585	7.515	7.660	2,105,031	2,000,000	0.20
1-Regular Grade	63	7.594	7.530	7.660	852,240	809,717	0.08
1-Mid Grade	58	7.593	7.475	7.655	1,295,322	1,230,692	0.12
1-Premium Grade	59	7.591	7.470	7.655	1,392,741	1,323,250	0.13
2-Regular Grade	64	7.589	7.530	7.655	486,391	462,122	0.05
2-Mid Grade	62	7.593	7.535	7.655	494,345	469,680	0.05
2-Premium Grade	55	7.591	7.530	7.655	690,231	655,792	0.07
2-Diesel	43	7.591	7.550	7.670	211,478	200,926	0.02
3-Regular Grade	64	7.587	7.465	7.650	1,127,090	1,070,854	0.11
3-Mid Grade	62	7.587	7.470	7.650	1,149,367	1,092,019	0.11
3-Premium Grade	52	7.587	7.520	7.645	1,028,558	977,238	0.10
3-Ethanol Free	44	7.585	7.520	7.645	905,763	860,570	0.09
Cumene Standard #2	1	7.570	7.490	7.650	5,943,042	2,000,000	0.20
4-Regular Unleaded	56	7.575	7.455	7.640	1,203,979	405,173	0.04
4-Mid Grade	59	7.576	7.460	7.640	1,223,605	411,777	0.04
4-Premium	58	7.574	7.455	7.635	1,305,727	439,414	0.04
4-Diesel	49	7.576	7.535	7.660	373,823	125,802	0.01
5-Regular Unleaded	62	7.573	7.455	7.640	1,405,543	473,005	0.05
5-Mid Grade	62	7.571	7.455	7.635	1,313,845	442,146	0.04
5-Premium	58	7.571	7.450	7.630	1,353,273	455,414	0.05
5-Diesel	49	7.569	7.530	7.650	549,469	184,912	0.02

## Supplementary Materials

The following are available online at https://www.mdpi.com/1660-4601/17/22/8380/s1, Figure S1: Chromatogram for Cumene Standard #1. Cumene appears as Peak #1 at 7.585 minutes, Figure S2: Chromatogram for 1-Regular Grade Gasoline. Cumene appears as Peak #63 at 7.594 minutes, Figure S3: Chromatogram for 1-Mid Grade Gasoline. Cumene appears as Peak #58 at 7.593 minutes, Figure S4: Chromatogram for 1-Premium Grade Gasoline. Cumene appears as Peak #59 at 7.591 minutes, Figure S5: Chromatogram for 2-Regular Grade Gasoline. Cumene appears as Peak #64 at 7.589 minutes, Figure S6: Chromatogram for 2-Mid Grade Gasoline. Cumene appears as Peak #62 at 7.593 minutes, Figure S7: Chromatogram for 2-Premium Grade Gasoline. Cumene appears as Peak #55 at 7.591 minutes, Figure S8: Chromatogram for 2-Diesel. Cumene appears as Peak #43 at 7.591 minutes, Figure S9: Chromatogram for 3-Regular Grade Gasoline. Cumene appears as Peak #64 at 7.587 minutes, Figure S10: Chromatogram for 3-Mid Grade Gasoline. Cumene appears as Peak #62 at 7.587 minutes, Figure S11: Chromatogram for 3-Premium Grade Gasoline. Cumene appears as Peak #52 at 7.587 minutes, Figure S12: Chromatogram for 2-Ethanol Free Gasoline. Cumene appears as Peak #44 at 7.585 minutes, Figure S13: Chromatogram Cumene Standard #2. Cumene appears as Peak #1 at 7.570 minutes, Figure S14: Chromatogram for 4-Regular Grade Gasoline. Cumene appears as Peak #56 at 7.575 minutes, Figure S15: Chromatogram for 4-Mid Grade Gasoline. Cumene appears as Peak #59 at 7.576 minutes, Figure S16: Chromatogram for 4-Premium Grade Gasoline. Cumene appears as Peak #58 at 7.574 minutes, Figure S17: Chromatogram for 4-Diesel. Cumene appears as Peak #49 at 7.576 minutes, Figure S18: Chromatogram for 5-Regular Grade Gasoline. Cumene appears as Peak #62 at 7.573 minutes, Figure S19: Chromatogram for 5-Mid Grade Gasoline. Cumene appears as Peak #62 at 7.571 minutes, Figure S20: Chromatogram for 5-Premium Grade Gasoline. Cumene appears as Peak #58 at 7.571 minutes, Figure S21: Chromatogram for 5-Diesel. Cumene appears as Peak #49 at 7.569 minutes.

## References

[1] Sivaranjani T., Xavier S., Periandy S. (2015). NMR, FT-IR, FT-Raman, UV spectroscopic, HOMO–LUMO and NBO analysis of cumene by quantum computational methods. J. Mol. Struct..

[2] National Toxicology Program (2013). Report on Carcinogens: Monograph on Cumene.

[3] O’Keefe W.F. (1984). Letter and Attachment to Toxic Substances Control Act Interagency Testing Committee.

[4] World Health Organization (WHO), Cumene (1999). Concise International Chemical Assessment Document 18.

[5] Glickman A.H., Alexander H.C., Buccafusco R.J., Morris C.R., Francis B.O., Suprenant D.C., Ward T.J. (1995). An evaluation of the aquatic hazard of cumene (isopropoyl benzene). Ecotoxicol. Environ. Saf..

[6] European Chemicals Agency (EHCA) (2019). Cumene: Substance Infracard.

[7] Florida Department of Environmental Protection (FDEP) Contaminated Site Cleanup. https://www.flrules.org/gateway/ChapterHome.asp?Chapter=62-780.

[8] Florida Department of Environmental Protection (FDEP) Contaminant Cleanup Target Levels. https://www.flrules.org/gateway/ChapterHome.asp?Chapter=62-777.

[9] Teply J., Dressler M. (1980). Direct determination of organic compounds in water using steam–solid chromatography. J. Chromatogr..

[10] Botta D., Castellani Pirri L., Mantica E., Angeletti G., Bjorseth A. (1984). Ground water pollution by organic solvents and their microbial degradation products. Analysis of Organic Micro-Pollutants in Water: Proceedings of the 3rd European Symposium, Oslo, Norway, 19–21 September 1983.

[11] Montz W.E., Puyear R.L., Brammer J.D. (1984). Identification and quantification of water-soluble hydrocarbons generated by two cycle outboard motors. Arch. Environ. Contam. Toxicol..

[12] Steurmer D.H., Ng D.J., Morris C.J. (1982). Organic contaminants in groundwater near an underground coal gasification site in northeastern Wyoming. Environ. Sci. Technol..

[13] National Library of Medicine (2005). Cumene. Hazardous Substances Database.

[14] U.S. Environmental Protection Agency (USEPA) (2017). How to Evaluate Alternative Cleanup Technologies for Underground Storage Tank Sites: A Guide for Corrective Action Plan Reviewers.

[15] Fontenot K.K., Bush E.W., Portier R.J., Meyer B.A., Beasley J.S., Walsh M.M. (2008). Determining an optimum tree species for the phytoremediation of Cumene and 4-Cumylphenol in groundwater. J. Environ. Hortic..

[16] Dutton G.D., Miller H.D. (1991). Georgia-Pacific successfully completes one of the first large-scale hazardous waste bioremediation projects in the southeastern United States. Proceedings HMC-South’91 Conference and Exhibition.

[17] Walker J.D., Austin H.F., Colwell R.R. (1975). Utilization of mixed hydrocarbon substrate by petroleum-degrading microorganisms. J. Gen. Appl. Microbiol..

[18] Walker J.D., Calomiris J.J., Herbert T.L., Colwell R.R. (1976). Petroleum hydrocarbons: Degradation and growth potential for Atlantic Ocean sediment bacteria. Mar. Biol..

[19] Mandelbaum R.T., Shati M.R., Ronen D. (1997). In situ microcosms in aquifer bioremediation studies. FEMS Microbiol. Rev..

[20] Acton D.W., Barker J.F. (1992). In situ biodegradation potential of aromatic hydrocarbons in anaerobic groundwaters. J. Contam. Hydrol..

[21] Beasley K.K., Grieg L.M., Suflita J.M., Nanny M.A. (2009). Polarizability and spin density correlate with the relative anaerobic biodegradability of alkylaromatic hydrocarbons. Environ. Sci. Technol..

[22] Dong X., Fushinobu S., Fukuda E., Terada T., Nakamura S., Shimizu K., Nojiri H., Omori T., Shoun H., Wakagi T. (2005). Crystal structure of the terminal oxygenase component of cumene dioxygenase from Pseudomonas fluorescens IP01. J. Bacteriol..

[23] U.S. Oil & Refining Co (2012). Material Safety Data Sheet: Gasoline. http://www.usor.com/files/pdf/4/Gasoline%20-%20SDS934%20120820.pdf.

[24] Canada Imperial Oil Limited, An Affiliate of Exxon Mobil Corporation (2009). Material Safety Data Sheet: Unleaded Gasoline for Export. http://www.nglenergypartners.com/wp-content/uploads/ExxonMobil.GasolineUnleadedEtOH_03.30.2009.pdf.

[25] Gulf Oil Limited Partnership (2018). Safety Data Sheet: Unleaded Gasoline, Non-Oxygenated, All Grades. https://msds.gulfoil.com/PDF/2018%20PDF/Gulf%20SDS%20Gasoline%20%20Non-Oxygenated.pdf.

[26] Sunoco, Inc. (2014). Safety Data Sheet: Sunoco Regular Gasoline with 10% Ethanol. https://static1.squarespace.com/static/51e0d2dde4b03a4e2198c2f0/t/55b6f6d1e4b05525c7002c5b/1438054097738/Gasoline+SDS.pdf.

[27] Citgo Petroleum Corporation (2018). Safety Data Sheet: Citgo Gasolines, All Grades Unleaded. http://www.docs.citgo.com/msds_pi/UNLEAD.pdf.

[28] Marathon Petroleum Company LP. (2018). Safety Data Sheet: Marathon Petroleum Gasolines-All Grades Unleaded. https://www.marathonbrand.com/content/documents/brand/sds/0127MAR019.pdf.

[29] Petrocom Energy Group, LLC (2008). Material Safety Data Sheet: Gasoline, MSDS No. PEG-UNL. http://msds.mkap.com/LiquidFuelProducts/UnleadedGasoline.pdf.

[30] Valero Marketing & Supply Company and Affiliates (2014). Safety Data Sheet: Unleaded Gasoline. https://www.marathonbrand.com/content/documents/brand/sds/0127MAR019.pdf.

[31] CRC Handbook of Chemistry and Physics. http://www.hbcponline.com/.

[32] Center for Disease Control and Prevention (CDC)-NIOSH Pocket Guide to Chemical Hazards—Cumene (2019). Centers for Disease Control and Prevention. https://www.cdc.gov/niosh/npg/npgd0159.html.

[33] Zambrano J., Stoner S. (1998). Ambient Water Quality Standards and Guidance Values and Groundwater Effluent Limitations. https://www.dec.ny.gov/docs/water_pdf/togs1112.pdf.

[34] Florida Department of Environmental Protection Groundwater and Surface Water Cleanup Target Levels. https://floridadep.gov/sites/default/files/2-GroundwaterandSurfaceWaterCleanupTargetLevels_3.pdf.

[35] State of Maryland Department of the Environment (2018). Cleanup Standards for Soil and Groundwater. https://mde.state.md.us/programs/LAND/MarylandBrownfieldVCP/Documents/www.mde.state.md.us/assets/document/MDESoilandGroundwaterCleanupStandards10-2018InterimFinalUpdate3-2.pdf.

[36] Delaware Department of Natural Resources and Environmental Control (1999). Remediation Standards Guidance Under the Delaware Hazardous Substance Cleanup Act. http://www.dnrec.state.de.us/DNREC2000/Divisions/AWM/sirb/DOCS/PDFS/Misc/RemStnd.pdf.

[37] North Carolina Department of Environmental Quality (2019). Subchapter 2L-Groundwater Classification and Standards. http://reports.oah.state.nc.us/ncac/title15a-environmentalquality/chapter02-environmentalmanagement/subchapterl/subchapterlrules.pdf.

[38] Minnesota Department of Health (2019). Comparison of State Water Guidance and Federal Drinking Water Standards. https://www.health.state.mn.us/communities/environment/risk/guidance/waterguidance.html.

[39] Maine Department of Environmental Protection (2018). Maine Remedial Action Guidelines (RAGs) for Sites Contaminated with Hazardous Substances. https://www.maine.gov/dep/spills/publications/guidance/rags/ME-Remedial-Action-Guidelines-10-19-18cc.pdf.

[40] Kansas Department of Health and Environment (2015). Risk-Based Standards for Kansas. http://www.kdheks.gov/remedial/download/RSK_Manual_15.pdf.

[41] Illinois Environmental Protection Agency (2012). Section 620.410 Groundwater Quality Standards for Class I: Potable Resource Groundwater. http://www.ilga.gov/commission/jcar/admincode/035/035006200D04100R.html.

[42] Iowa Department of Natural Resources (2016). Statewide Standards for a Protected Groundwater Source, Statewide Standards-Cumulative Risk Calculator. https://programs.iowadnr.gov/riskcalc/Home/statewidestandards.

[43] New Jersey Department of Environmental Protection (2019). Ground Water Quality Standards-Class IIA by Constituent. https://www.nj.gov/dep/standards/groundwater.pdf.

[44] Michigan Department of Environmental Quality (2019). Part 22 Groundwater Quality-Standards List. https://www.michigan.gov/documents/deq/wrd-groundwater-p22-standards_564955_7.pdf.

[45] New Hampshire Department of Environmental Services (2018). NHDES Risk Characterization and Management Policy (Section 7.4)-Table 2, Method 1 Groundwater Standards. https://www.des.nh.gov/organization/divisions/waste/hwrb/documents/rcmp.pdf.

[46] Pennsylvania Department of Environmental Protection (2011). Standards/Action Levels for Confirmatory Samples Collected at Closure Site Assessments. http://files.dep.state.pa.us/EnvironmentalCleanupBrownfields/StorageTanks/StorageTanksPortalFiles/attachment-5.pdf.

[47] Ladino-Orjuela G., Gomes E., Silva R.D., Salt C., Parsons J.R. (2016). Metabolic pathways for degradation of aromatic hydrocarbons by bacteria. Rev. Environ. Contam. Toxicol..

[48] Suthersan S., Houston K., Schnobrich M., Horst J. (2011). Engineered anaerobic bio-oxidation systems for petroleum hydrocarbon residual source zones with soluble sulfate application. Groundw. Monit. Remediat..

[49] Wiedermeier T.H., Rifai H.S., Newell C.J., Wilson J.T. (1999). Natural Attenuation of Fuels and Chlorinated Solvents in the Subsurface.

[50] Hussain A., Hasan A., Javid A., Qazi J.I. (2016). Exploited application of sulfate-reducing bacteria for concomitant treatment of metallic and non-metallic wastes: A mini review. 3 Biotech.

[51] United States Environmental Protection Agency (USEPA) (2018). 2018 Edition of the Drinking Water Standards and Health Advisories Tables, EPA 822-F-18-001. https://www.epa.gov/sites/production/files/2014-03/documents/tum_ch1.pdf.

[52] United States Environmental Protection Agency (USEPA) (1996). Method 8260B Volatile Organic Compounds by Gas. Chromatography/Mass Spectometry (GC/MS). https://19january2017snapshot.epa.gov/sites/production/files/2015-12/documents/8260b.pdf.

[53] U.S. Environmental Protection Agency (USEPA) Toxicological Review of Cumene. In Support. of Summary Information on the Integrated Risk Information System. June 1997. https://cfpub.epa.gov/ncea/iris/iris_documents/documents/toxreviews/0306tr.pdf.

[54] Hazardous Substances DataBank (HSDB) (2017). Isopropylbenzene. https://toxnet.nlm.nih.gov/.

[55] Occupational Safety and Health Administration (OSHA) (2015). OSHA Annotated Table Z-1.

[56] Florida Department of Health Environmental Health Bureau of Environmental Health, Water Programs Maximum Contaminant Levels and Health Advisory Levels. http://www.floridahealth.gov/environmental-health/drinking-water/_documents/hal-list.pdf.

[57] U.S. Environmental Protection Agency (USEPA) (2020). Regional Screening Levels Generic Tables. https://www.epa.gov/risk/regional-screening-levels-rsls-generic-tables.

[58] U.S. Environmental Protection Agency (USEPA) (2018). 2018 Edition of the Drinking Water Standards and Health Advisories, March 2018.

[59] U.S. Environmental Protection Agency (USEPA) Vapor Intrusion Screening Level (VISL) Calculator Version 3.5 for June 2017 RSLs. https://www.epa.gov/vaporintrusion/vapor-intrusion-screening-level-calculator.

[60] International Agency for Research on Cancer (IARC) (2013). Some Chemicals Present in Industrial and Consumer Products, Food, and Drinking Water.

[61] U.S. Environmental Protection Agency (USEPA) (2014). Vapor Intrusion Screening Level (VISL) Calculator User’s Guide. https://www.epa.gov/vaporintrusion/vapor-intrusion-screening-level-calculator.

