# Supplementary material for: Cumene Contamination in Groundwater: Observed Concentrations, Evaluation of Remediation by Sulfate Enhanced Bioremediation (SEB), and Public Health Issues
Source: Int J Environ Res Public Health. 2020 Nov 12;17(22):8380. doi: 10.3390/ijerph17228380 (PMC7696069; doi:10.3390/ijerph17228380)
Supplement: Supplementary file 1 [file ijerph-17-08380-s001.pdf]

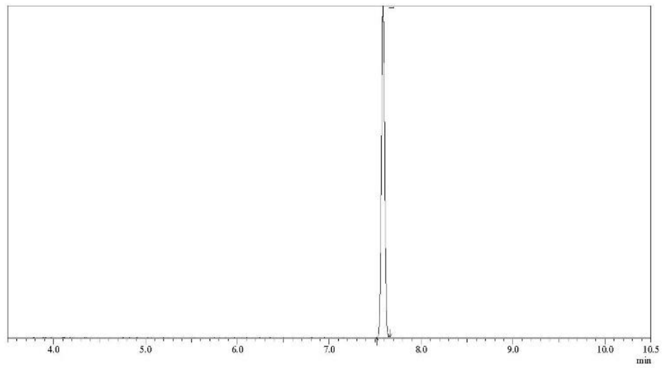

Figure S1. Chromatogram for Cumene Standard #1. Cumene appears as Peak #1 at 7.585 minutes

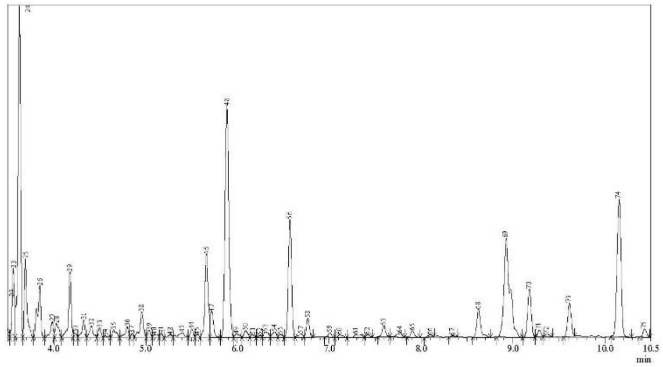

Figure S2. Chromatogram for 1-Regular Grade Gasoline. Cumene appears as Peak #63 at 7.594 minutes

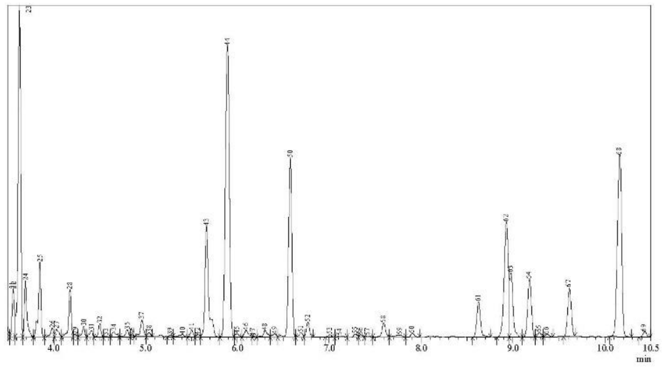

Figure S3. Chromatogram for 1-Mid Grade Gasoline. Cumene appears as Peak #58 at 7.593 minutes

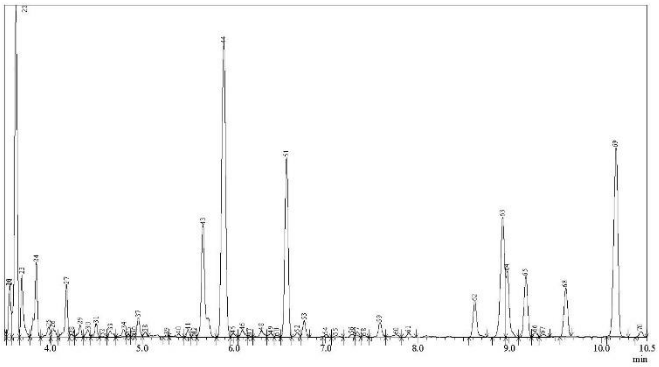

Figure S4. Chromatogram for 1-Premium Grade Gasoline. Cumene appears as Peak #59 at 7.591 minutes

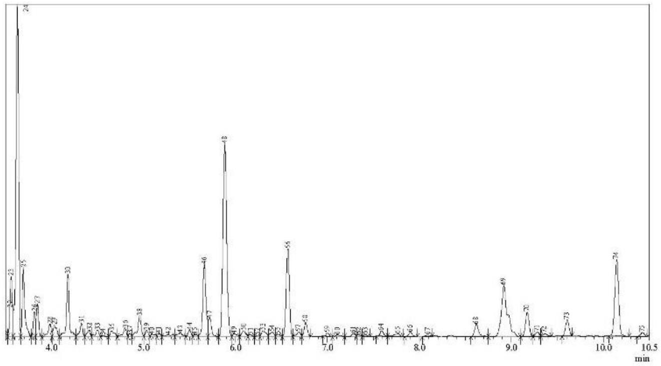

Figure S5. Chromatogram for 2-Regular Grade Gasoline. Cumene appears as Peak #64 at 7.589 minutes

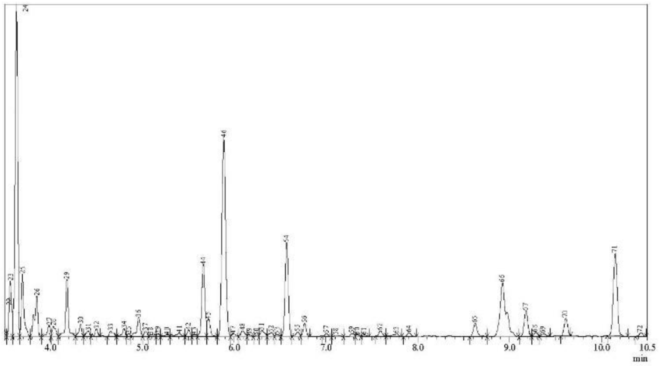

Figure S6. Chromatogram for 2-Mid Grade Gasoline. Cumene appears as Peak #62 at 7.593 minutes

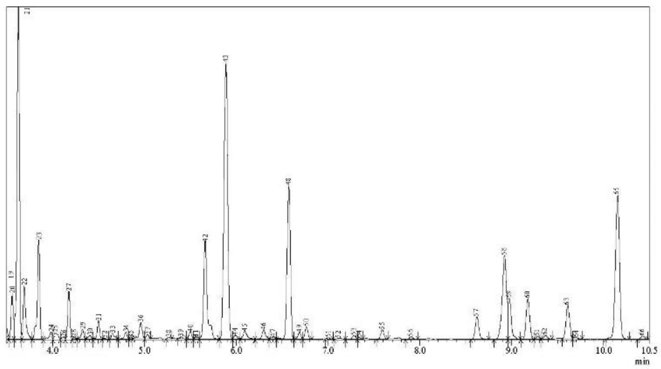

Figure S7. Chromatogram for 2-Premium Grade Gasoline. Cumene appears as Peak #55 at 7.591 minutes

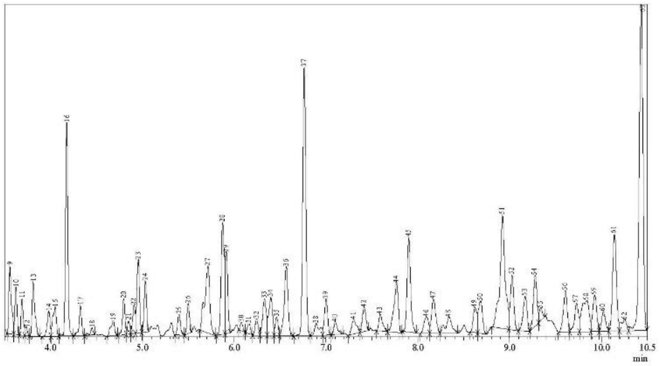

Figure S8. Chromatogram for 2-Diesel. Cumene appears as Peak #43 at 7.591 minutes

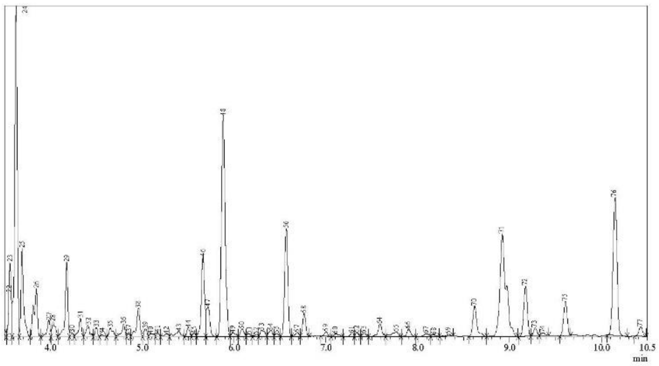

Figure S9. Chromatogram for 3-Regular Grade Gasoline. Cumene appears as Peak #64 at 7.587 minutes

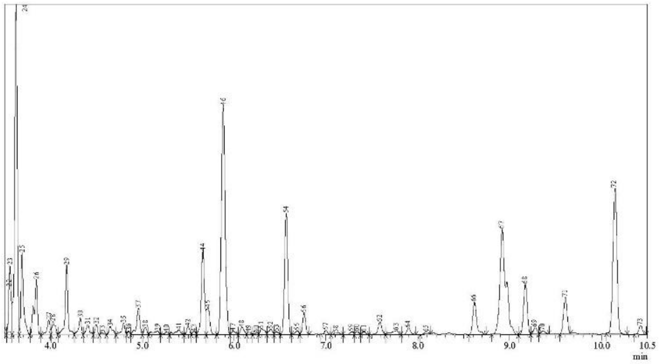

Figure S10. Chromatogram for 3-Mid Grade Gasoline. Cumene appears as Peak #62 at 7.587 minutes

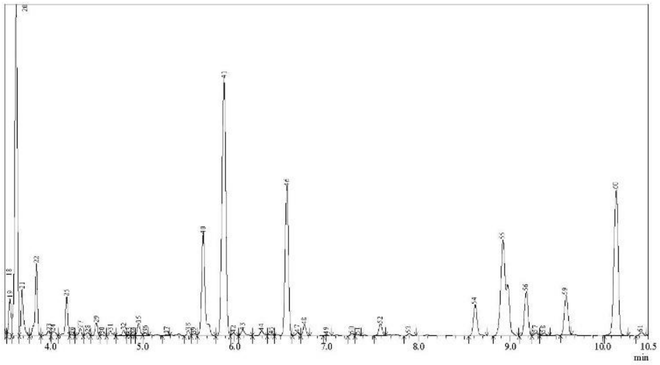

Figure S11. Chromatogram for 3-Premium Grade Gasoline. Cumene appears as Peak #52 at 7.587 minutes

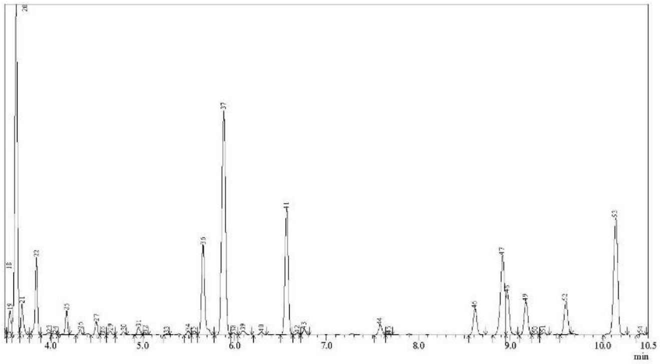

Figure S12. Chromatogram for 2-Ethanol Free Gasoline. Cumene appears as Peak #44 at 7.585 minutes

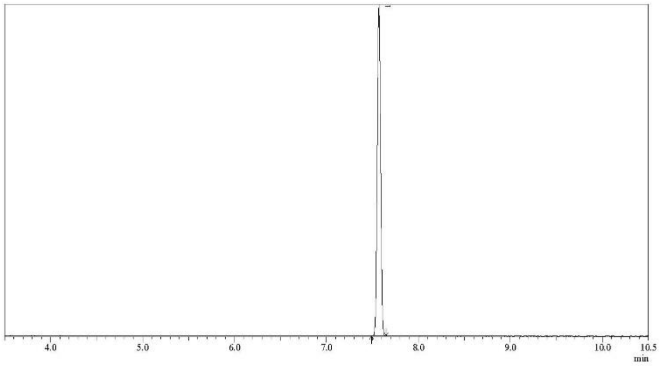

Figure S13. Chromatogram Cumene Standard #2. Cumene appears as Peak #1 at 7.570 minutes

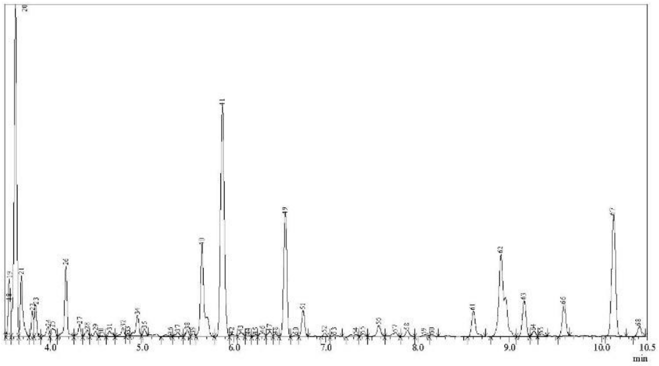

Figure S14. Chromatogram for 4-Regular Grade Gasoline. Cumene appears as Peak #56 at 7.575 minutes

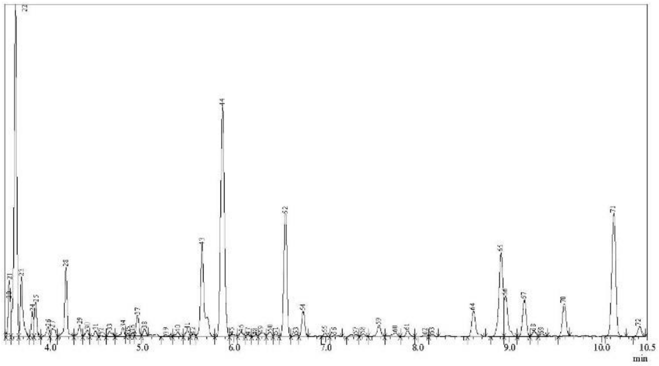

Figure S15. Chromatogram for 4-Mid Grade Gasoline. Cumene appears as Peak #59 at 7.576 minutes

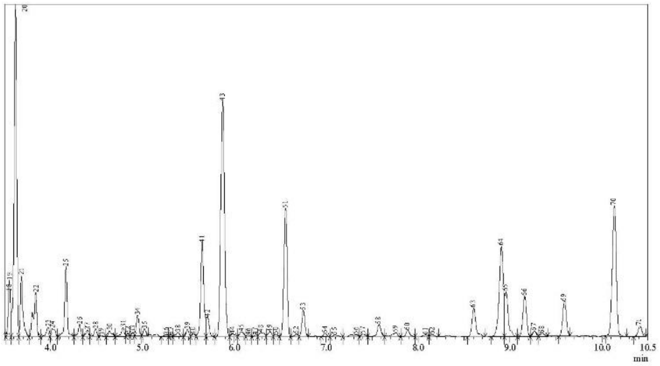

Figure S16. Chromatogram for 4-Premium Grade Gasoline. Cumene appears as Peak #58 at 7.574 minutes

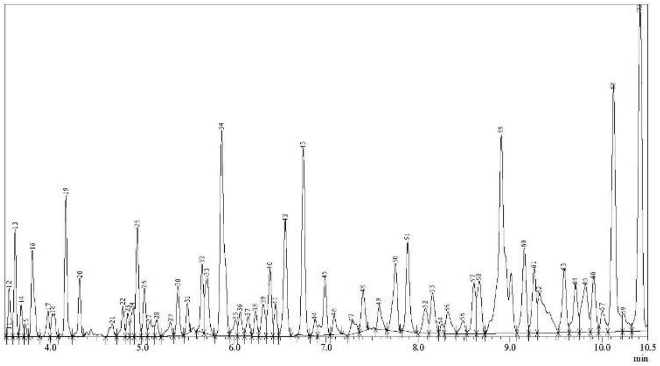

Figure S17. Chromatogram for 4-Diesel. Cumene appears as Peak #49 at 7.576 minutes

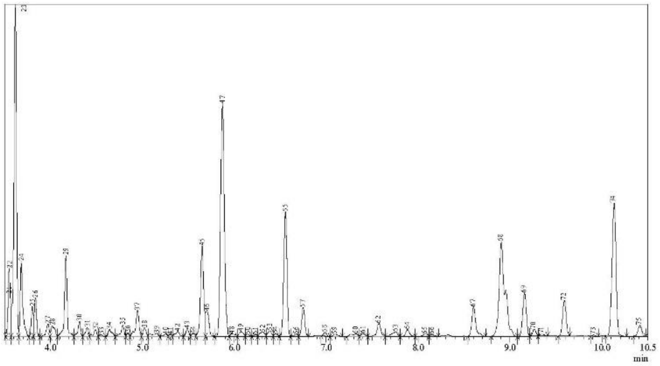

Figure S18. Chromatogram for 5-Regular Grade Gasoline. Cumene appears as Peak #62 at 7.573 minutes

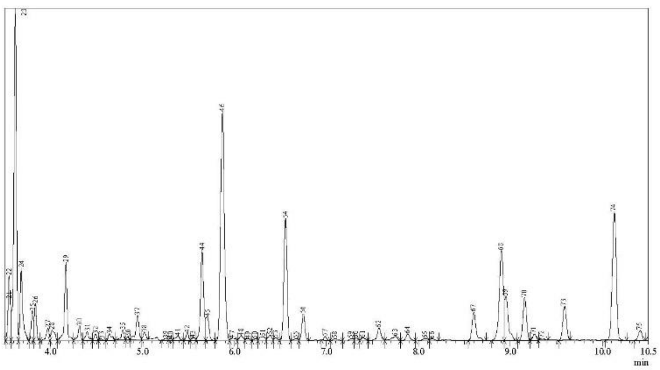

Figure S19. Chromatogram for 5-Mid Grade Gasoline. Cumene appears as Peak #62 at 7.571 minutes

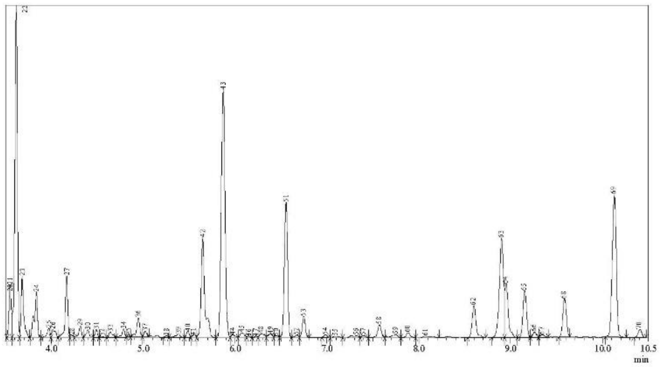

Figure S20. Chromatogram for 5-Premium Grade Gasoline. Cumene appears as Peak #58 at 7.571 minutes

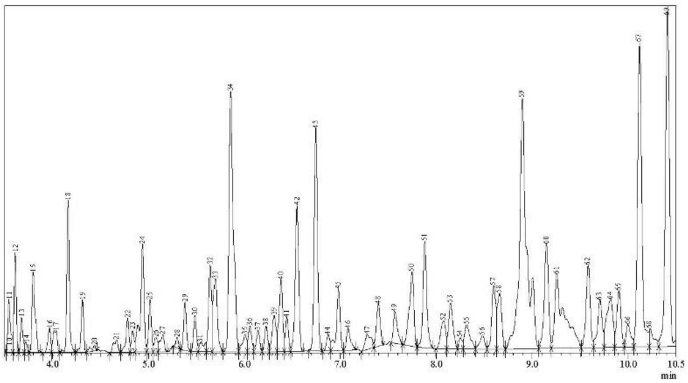

Figure S21. Chromatogram for 5-Diesel. Cumene appears as Peak #49 at 7.569 minutes
